# Supplementary material for: Dynamic properties of noise and Her6 levels are optimized by miR‐9, allowing the decoding of the Her6 oscillator
Source: EMBO J. 2020 May 12;39(12):e103558. doi: 10.15252/embj.2019103558 (PMC7298297; doi:10.15252/embj.2019103558)
Supplement: Supplementary file 1 — Appendix [file EMBJ-39-e103558-s001.pdf]

TABLE OF CONTENTS

Appendix Figure S1-S5

Appendix Table S1-S2

Appendix Figure Legend S1-S5

Appendix Table Legend S1-S2

Appendix Figure S1

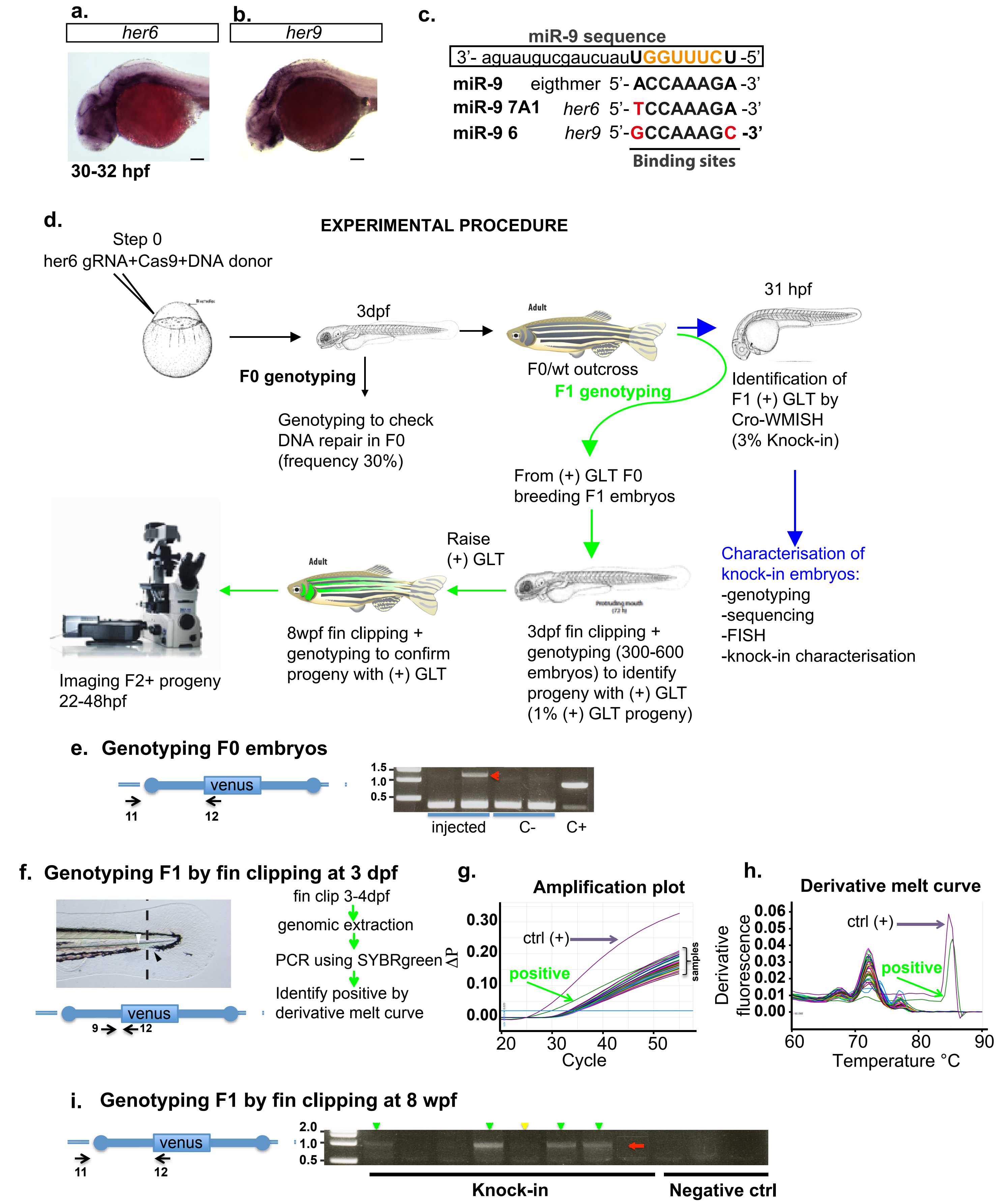

Appendix Figure S2

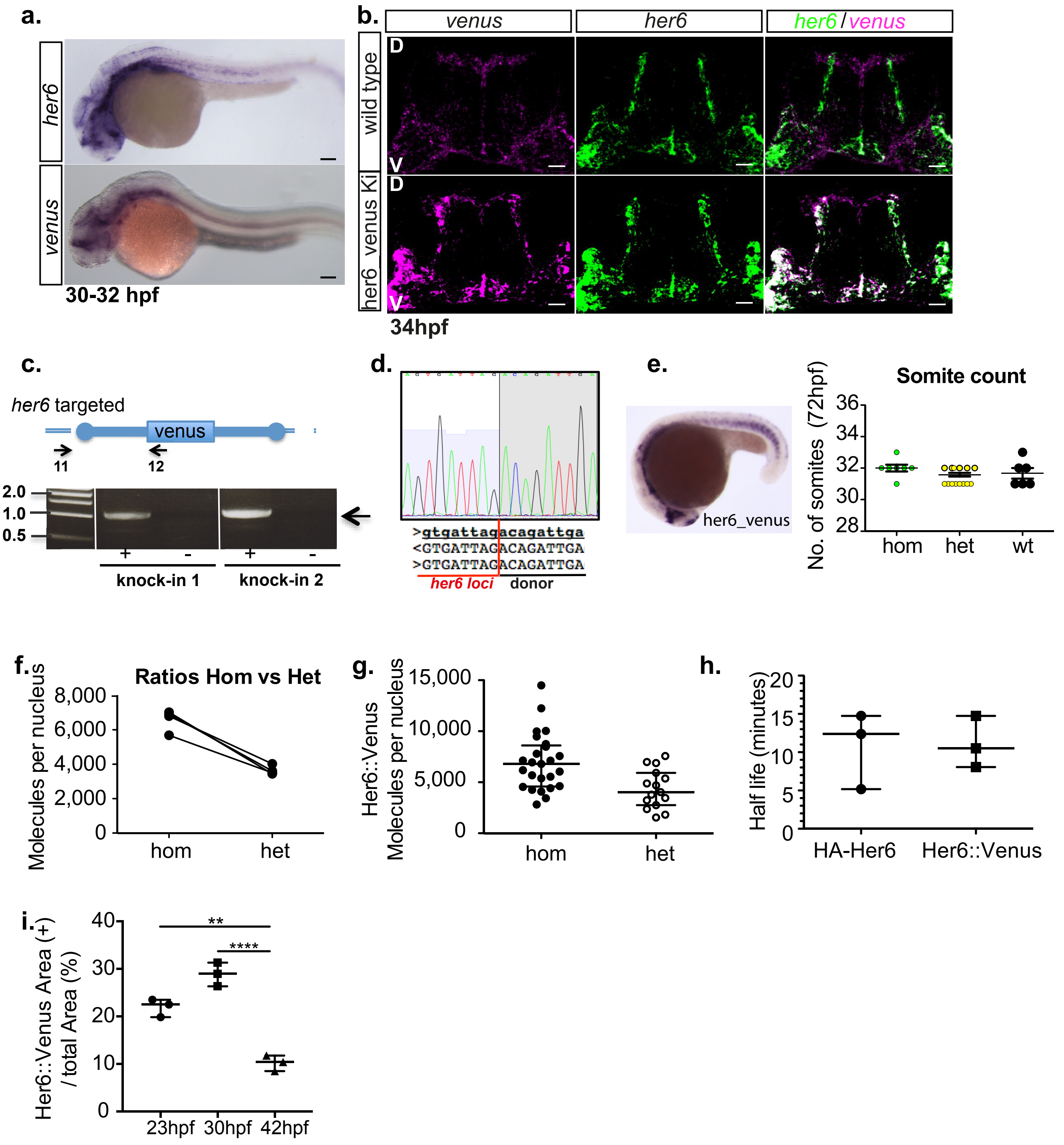

Appendix Figure S3

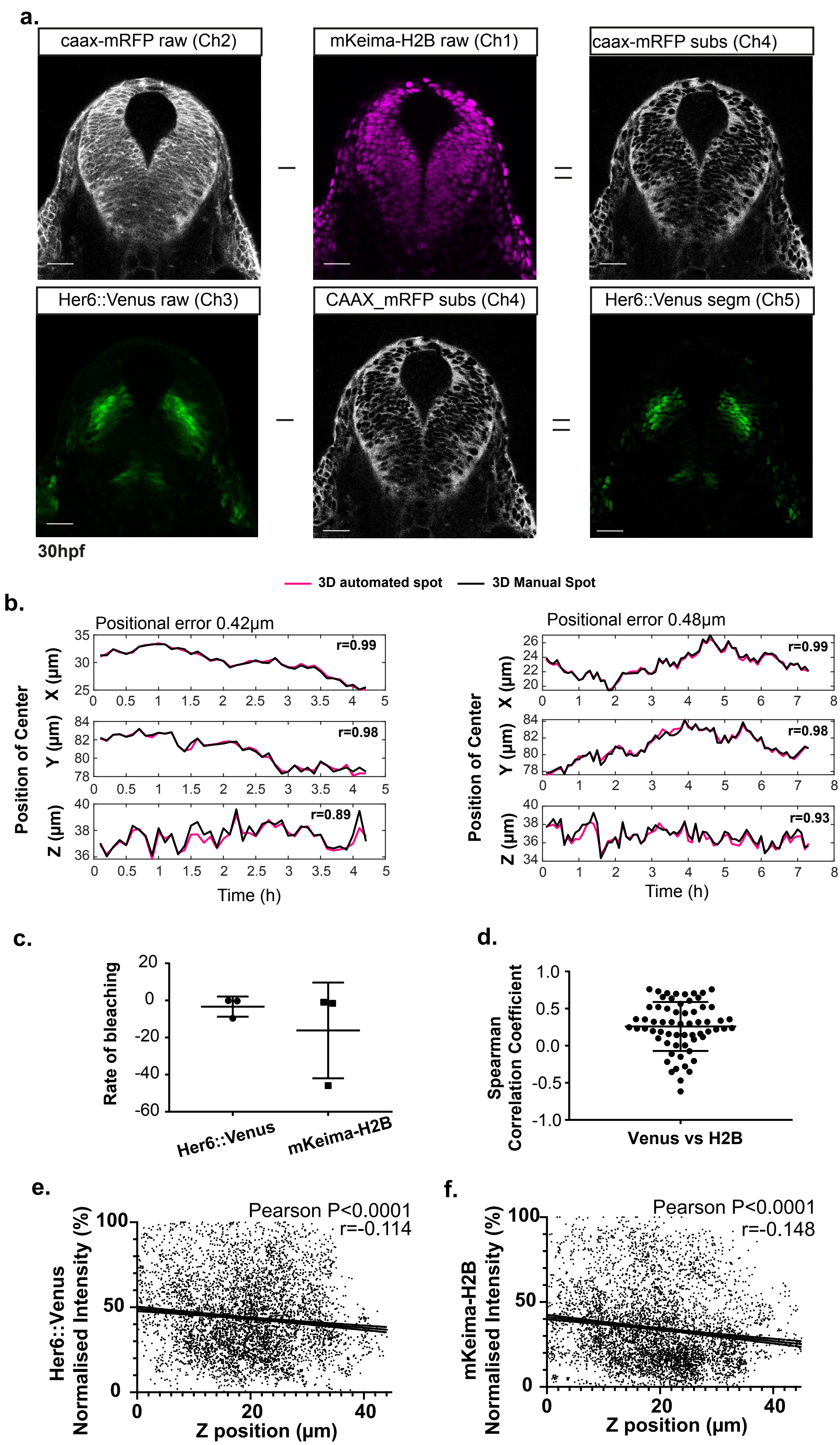

Appendix Figure S4

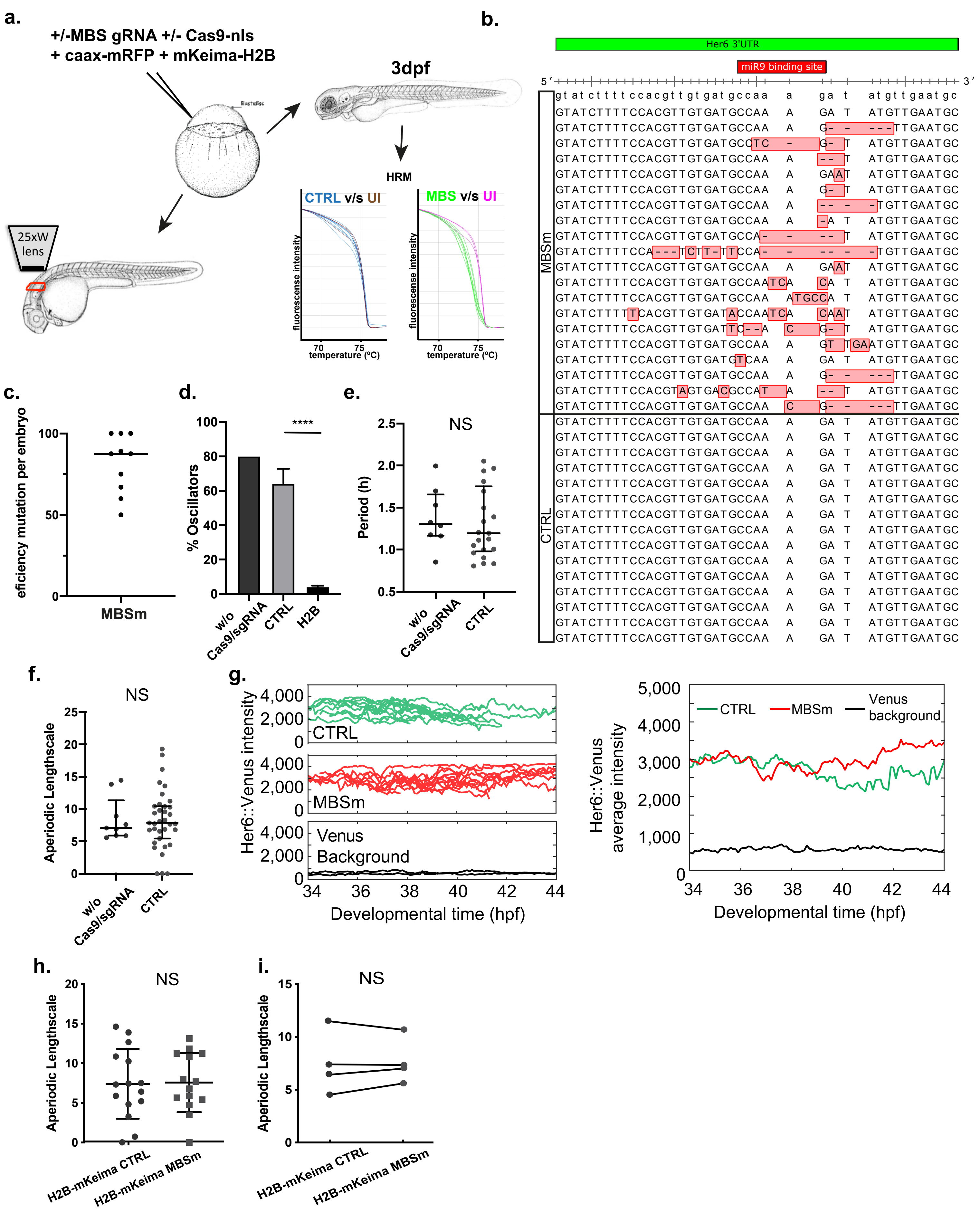

Appendix Figure S5

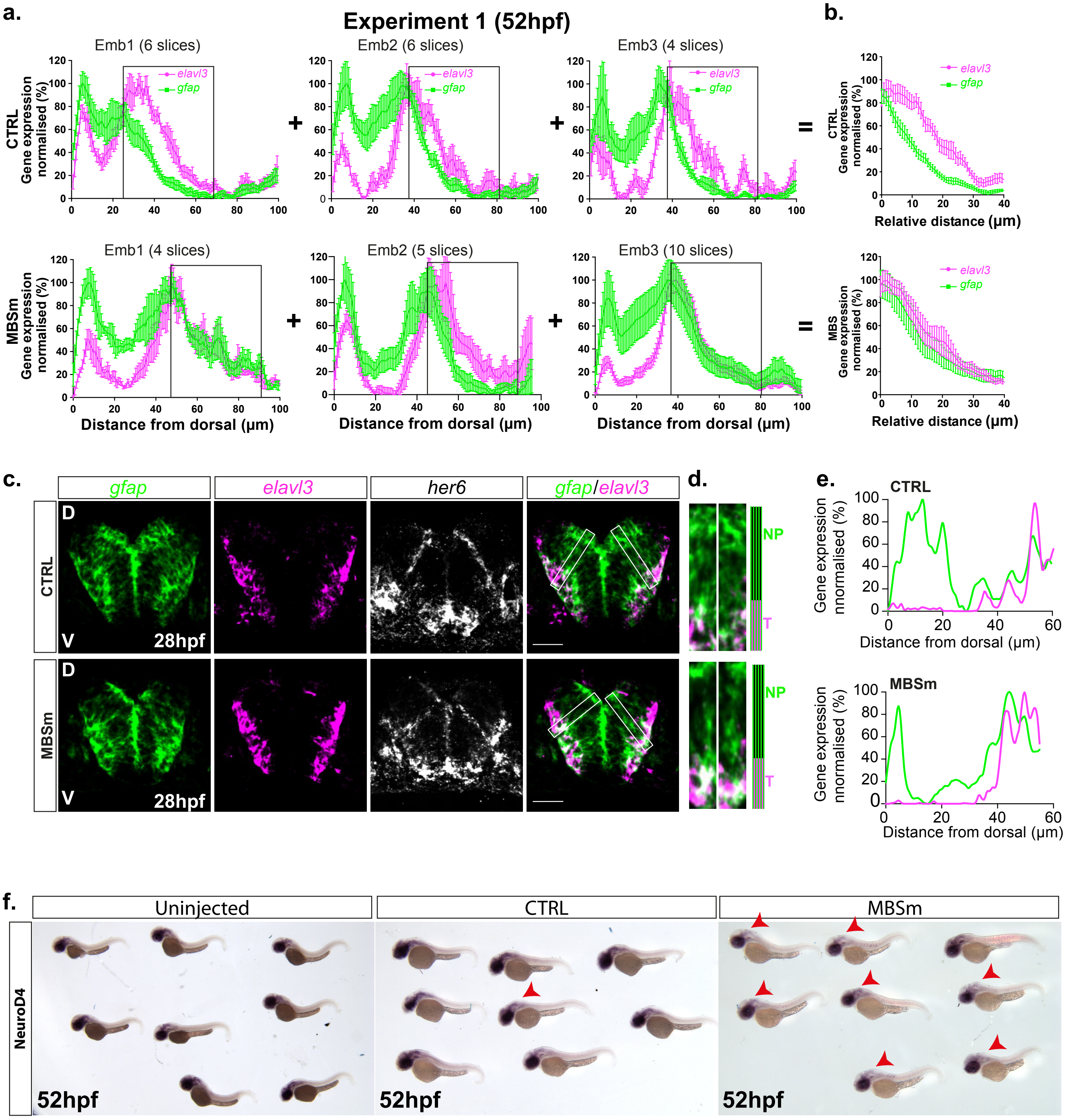

Appendix Table S1.

No.	NAME	Sequence 5'-3'
1	her6 LA cloning forward	ggatccggcggccttggtagactccgagggaactatagtgcttcaagaagtgattag
2	her6 LA cloning reverse	agtcgaccctccactacctccccaaggccgccaaacggagtctgacg
3	Her6 RA cloning forward	tggtaccactccgaggagaaaaaaaaactcttaaaagac
4	her6 RA cloning reverse	ggcggccttggtagactccgaggccgcagtgtcagaaacaccgtatattaacatg
5	Linker_venus cloning forward	agggtcgactgctagcggtggagtgagcaagg
6	Linker_venus cloning reverse	aaggtaccttacttgtacagctcgtccatgccg
7	her6 CRISPR sgRNA forward	tagg**ggcggccttggtagactccg**
8	her6 CRISPR sgRNA reverse	aaac**cggagtctaccaaggccgcc**
9	her6 CRISPR HRM forward	gtctacgcaaacaattccaa
10	her6 CRISPR HRM reverse	cgctgaacaaagaaaacaagt
11	her6 CRISPR genotyping LA forward	atgcctgccgatatcatgga
12	her6 CRISPR genotyping LA reverse	tgaacagctcctcgcccttg
13	gfap probe cloning forward	attctcctccaccatggag
14	gfap probe cloning reverse	agtgaaggagatcttcttctg
15	elavl3 probe cloning forward	gtgcatcttcgtctacaacctg
16	elavl3 probe cloning reverse	acagataactgcatgtggtgg
17	neurod4 cloning probe forward	agtgaggcacgagacgcgctctg
18	neurod4 cloning probe reverse	ttattcgctgtaaatgctgctc
19	her6 probe cloning forward	tcgtcgacaagatgcctgccgatatcatgg
20	her6 probe cloning reverse	ccaaggccgccaaacggagtctgacg
21	venus probe cloning forward	agtggagggtcgactgctagcggtggc
22	venus probe cloning reverse	aaggtaccttacttgtacagctcgtccatgccg
23	Pri-miR-9-4 probe cloning forward	ttccacaagggtatcgatag
24	Pri-miR-9-4 probe cloning reverse	tattatatgagaaccacgtg
25	her6 CRISPRscan sgRNA MBS mutation	taatacgactcactatagggcgcattcaacatatcttgttttagagctagaa
26	her6 CRISPRscan sgRNA MBS control	taatacgactcactataggatctttggcatcacaacggttttagagctag aa
27	her6 MBS mutation HRM forward	tgctgtagtgtggaaccactag
28	her6 MBS mutation HRM reverse	tgtattgttgaattccgttcaatgc
29	mKeima BamHi cloning forward	cgggatccaccggtcgccaccatg
30	mKeima BspeI cloning reverse	aattccggaaccgagcaaagagtggcgtg

Appendix Table S2

Probe sequence (5'-3')

	her6 smFISH probes	*elavl3* smFISH probes
**1**	tccatgatatcggcaggcat	tgctgttgtgtgtgtatttc
**2**	gaccggagaagaggagtttt	agaaaagcagtgatccccag
**3**	tgggtttatcaggtgtagtg	gtgggcgatggttcagaaaa
**4**	agactttctgtgttccgaag	gagagagagatttctctccg
**5**	tcttttctccataatgggtt	gggttttcttcaggatgatg
**6**	tttcgttgattctcgctctt	cagcagtgtgaaaatgagga
**7**	attaacgttttcagctgacc	ccgtgcgattgtaggttttc
**8**	atcttttttcagagcatcca	aaaaggacagtcttcgccag
**9**	ggctttctcaagtttagagt	gcaaatgtcagtttccaaag
**10**	tcactgtcatctccaggatg	ccgtggcttggaatggagaa
**11**	cgctgcatgtttctgagatg	caggctggattgcagatatt
**12**	tttagggcagcggtcatttg	gatctaggtccttcgatttg
**13**	ttcccaagaacggtgggatc	ccattcttgacgtacaaaga
**14**	attcactgaatccagctcgg	tccatggtgctaattatagt
**15**	aaccgggtaacctcgttcat	cggaccattggacacctgag
**16**	tgttaaccccttcacatgtg	gttagtgctaatgacagggc
**17**	cgttgatctgtgtcatgcag	ttttgctgtcatctgtggcg
**18**	tgctgtgttggatagttcat	aggtagttgacgatcaggtt
**19**	tgaaggatggatgaggaggc	ttcctgggtcatgttctgag
**20**	gggatctgaaccatgggttg	tgccaaagaggctcttgaac
**21**	cgctaagaggcacaacgttg	atttgcaggactcgatttcc
**22**	gtcaaattggaggatgagcc	cctgtgatcttgtctctgac
**23**	ccatatactttagttgcgtc	tacaaagccatatcccaagc
**24**	ttgccggcacaagctggaaa	cgtcgttgggatccacatag
**25**	caaaaaggcgaactgtccgt	tgagcgtgttgatagccttg
**26**	ttggagcaaaggcagcgttg	tttggtctgcagtttgagac
**27**	tagactggaataacagggcc	tggcgtaagacaccttgatt
**28**	gaaccggtgtgttggaattg	
**29**	aaacggagtctgacgtgacg	

Appendix Figures Legend

Appendix Figure S1. Generation of the Zebrafish knock-in reporter. Related to Figure 1. (a-b) Chromogenic whole mount in situ hybridization (Cro-WMISH) for *her6* **(a)** and *her9* **(b)**; longitudinal view, scale bar 100µm. **(c)** *her6* and *her9* miR-9 binding site sequence vs complementary eigthmer sequence of miR-9 with mutations shown in red. Box shows the miR-9 sequence with binding site in bold capital. **(d)** Diagram of experimental approach used to generate the Her6::Venus knock-in. F0: founder generation 0, GLT: germ line transmission, F1: Founder generation 1, F2: Founder generation 2, wt: wild type, FISH: Fluorescent in situ hybridization **(e)** Amplicon generated by using primers 11 and 12, indicated by red arrowhead, obtained only when *venus* is inserted at C-terminus of *her6* gene. C-: uninjected embryos; C+: Her6::venus plasmid used as positive control. (**f-h)** Genotyping by fin clipping at 3dpf, qPCR amplification **(g),** followed by identification of positive fish by derivative of melt curve **(h);** ctrl (+) denotes positive PCR control; positive knock-in is marked by green arrow). **(i)** Representative example of genotyping by fin clipping at 8wpf.

Appendix Figure S2. Characterization of the Zebrafish knock-in reporter. Related to Figure 1. (a) Chromogenic WM-ISH for *her6* in wildtype embryos (top), and *venus* in Her6::Venus knock-in embryos (bottom); 30-32hpf; longitudinal view, anterior to the left, scale bar 100µm. **(b)** Transverse sections of double fluorescent WM-ISH for *her6* (green) and *venus* (magenta) observed in wild type embryo (top panel) and Her6::Venus knock-in embryo (bottom panel) at 34hpf showing agreement between *her6* and *venus* expression; scale bar 20µm; anterior (A), posterior (P),dorsal (D), ventral (V), otic vesicle (ov). **(c)** Amplicon generated by using primers 11 and 12 following schematic **(Figure 1b)**, obtained only when Venus is inserted at C-terminus of *her6* gene; Her6::Venus knock-in embryo(+), wildtype sibling embryo(-). Knock-in 1 and 2 are two different founder animals. **(d)** Sequencing showing the correct DNA repair in exon1 of *her6* gene. **(e)** Chromogenic WM-ISH for *venus* in Her6::Venus knock-in at 18 somites development (left); comparison of number of somites observed in homozygote, heterozygote and wild type embryos at 72hpf (right); bars represent mean and SD of (hom: 7 embryos), (het: 19 embryos) and (wt: 6 embryos). **(f)** Pairwise comparison of protein abundance in the hindbrain of homozygous versus heterozygous embryos containing the Her6::Venus knock-in; data represents median per experiment from (hom: 4 embryos, 90 cells), (het: 4 embryos, 72 cells). **(g)** Representative example of Her6::Venus protein abundance observed in the hindbrain (r6) of homozygous versus heterozygous embryos; 34±1hpf, bars represent median and interquartile range of (hom: 1 embryo, 25 cells) and (het: 1 embryo, 15 cells). **(h)** Half-life of HA-Her6 protein versus

Her6::Venus protein measured in vitro by injection of mRNA in embryos; bars represent median and interquartile range from 3 biological repeats. **(i)** Quantification of Her6::Venus(+) area during development; bars indicate median and interquartile range of counts collected from 3 z-stacks per embryo and one embryo per condition; one-way ANOVA with Bonferroni multiple comparison test with significance p<0.01**.

Appendix Figure S3. Pre-processing of video and single cell tracking data. Related to Figure 2. (a) Raw images of hindbrain r6 30hpf showing the membrane marker, caax-mRFP (grey), the nuclear marker, mKeima-H2B (magenta) and Her6::Venus (green); image processing steps included subtraction of nuclear from membrane marker to generate a caax-mRFP subtracted channel; this is then subtracted from Venus to produce a Her6::Venus segmented channel with enhanced separation between nuclei; scale bar 30μm. **(b)** Examples of center of mass X, Y and Z coordinates determined by 3D automated Imaris tracking versus 3D manual spot tracking performed independently in the same nucleus used to quantify average positional error; r denotes Spearman rank correlation values computed between manual and automated position corresponding to the X, Y and Z positions; left and right panels indicate results from separate tracks. Black traces: 3D manual spot tracking. Red traces: 3D automated spot tracking **(b)** Rate of bleaching observed as mean per experiment in the Venus and mKeima channels; error bars represent mean and SD per condition. **(c)** Spearman rank correlation coefficient calculated from Her6::Venus versus mKeima-H2B time series observed in the same cell; bars represent median and interquartile range of 35 cells, n=3 embryos. **(d)** Visualisation of Her6::Venus intensity normalized per experiment versus z-position in all data collected; statistical p-value obtained from Pearson linear correlation test. **(e)** Visualisation of mKeima-H2B intensity normalized per experiment versus z-position in all data collected; statistical p-value obtained from Pearson's linear correlation test.

Appendix Figure S4. MiR-9 binding site manipulation (MBSm) and dynamic data analysis of WT, CTRL and MBSm. Related to Figures 2 to 4. (a-left) Schematic representation of experimental procedure used to mutate the Her6 miR-9 binding site; **(a-right)** high resolution melt graph obtained from: control (CTRL-blue) versus uninjected (UI-brown); and MBS mutation (MBSm-green) versus uninjected (UI-magenta). **(b)** Representative examples of sequences obtained from F0 embryos showing a mutation in the miR-9 binding site (MBSm) and no mutation obtained from CTRL embryos. **(c)** Efficiency of mutation per embryo calculated from a total of 10 MBSm embryos with 8 sequences on average per embryo. **(d)** Proportions of oscillators detected from Her6::Venus/H2B timeseries

collected from Her6::Venus knock-in embryos injected without Cas9nls and sgRNA (w/o Cas9/sgRNA), also shown in **Figure 2f-**34hpf) versus knock-in embryos injected with Cas9nls and sgRNA that does not induce mutations (CTRL, also shown in **Figure 4c**) and corresponding H2B timeseries; error bars represent mean with SD of 4 experiments per condition; statistics indicate two-tailed Student t test with p<0.0001****. **(e)** Period estimated from Her6::Venus/H2B timeseries collected from w/o Cas9/sgRNA embryos (also shown in **Figure 2g**-34hpf) versus CTRL Her6::Venus/H2B (also used in **Figure 4d**) and corresponding mKeima-H2B observed in the same cells. **(f)** Aperiodic lengthscale values estimated from Her6::Venus/H2B of w/o Cas9/sgRNA (also shown in **Figure 2h**) versus CTRL (also used in **Figure 4j**) embryos at 34hpf. **(e-f)** Analysis used (w/o Cas9/sgRNA: 10 cells, 1 embryo) and (CTRL: 40 cells, n=4) embryos at 34hpf; bars indicate median with interquartile range; Mann-Whitney two-tailed test not significant p=0.55 **(e)**, p=0.86 **(f)**. **(g)** Single cell time series and average of Her6::Venus intensities observed in a paired experiment showing CTRL (15 cells, 1 embryo) versus MBSm (14 cells, 1 embryo); Venus background represents technical noise observed in areas of the tissue without expression of the fluorophore. **(h)** Comparative analysis of aperiodic lengthscale quantified from mKeima-H2B timeseries in paired CTRL versus MBSm embryos at 34hpf; bars indicate median with interquartile range; Mann-Whitney two-tailed test not significant p=0.88. **(i)** Pairwise comparison of aperiodic lengthscale from mKeima-H2B; markers indicate median per experiment from CTRL (15 cells, 5 cells, 13 cells, 7 cells; n=4) and MBSm (14 cells, 5+12 cells, 4+8 cells, 13 cells; n=6) embryos at 34hpf; paired Student t test not significant p=0.66.

Appendix Figure S5. Effect of miR-9 binding site manipulation (MBSm) on expression of Her6 and downstream targets. Related to Figure 6. (a) Example of how quantitative analysis was done on *gfap* (green) and *elavl3* (magenta) across the dorsal to ventral axis of the Her6 domain observed in Control and MBSm embryos at 52hpf; data represents mean with SD of 4-10 slices per embryo; The region of interest (ROI) is delimitated by positioning the black box in the intersection of the highest expression of *elavl3* and *gfap.* This allowed us to identify T zone characterised by overlapping high expression of *elavl3* and *gfap* (*gfap*(+)/*elavl3*(+)) and N zones characterised by high elavl3 and low *gfap* (*gfap*(-)/*elavl3*(+)). **(b)** Graphic representation of the average *gfap* and *elavl3* intensity distribution across multiple embryos in the ROI described in **(a).** CTRL embryos show only at the intersection (at 0μm in the relative distance) *gfap*(+)/*elavl3*(+), T zone, and as relative distance increases it become *gfap*(-)/*elavl3*(+), N zone. MBSm embryos show only *gfap*(+)/*elavl3*(+), T zone. **(c)** Triple WM-FISH for *gfap* (green), *elavl3* (magenta) and *her6* (grey) in CTRL (top) and MBSm (bottom) embryos at 28hpf; transversal view; scale bar 30μm; annotations denote dorsal (D) and ventral

(V). **(d)** Magnification of inset from merged *gfap/elavl3* in **(a)** showing CTRL (top panel) and MBSm (bottom panel); annotations represent neural progenitor zone (NP=*gfap(+)/elavl3(-))* and transition zone (T=*gfap(+)/elavl3(+)*). **(e)** Normalized intensity mean of *elavl3* and *gfap at 28hpf* distributed across dorsal to ventral axis corresponding to **(d)** shows similar intensity profile in CTRL (top) and MBSm (bottom). **(f)** Chromogenic WM-ISH of neuroD4 observed in multiple uninjected, CTRL and MBSm embryos at 52hpf; anterior to the left. Red head-arrows show abnormal or weak neuroD4 expression when compared to CTRL or Un-injected.

Appendix Tables Legend

Appendix Table S1. Oligonucleotide sequences used for cloning, sgRNA, High Resolution Melt (HRM) and Genotyping.

Appendix Table S2. smFISH Probe sequences for *her6* and *elavl3.*
