# Supplementary material for: Dynamic properties of noise and Her6 levels are optimized by miR‐9, allowing the decoding of the Her6 oscillator
Source: EMBO J. 2020 May 12;39(12):e103558. doi: 10.15252/embj.2019103558 (PMC7298297; doi:10.15252/embj.2019103558)
Supplement: Supplementary file 2 — Expanded View Figures PDF [file EMBJ-39-e103558-s002.pdf]

Expanded View Figures

Figure EV1. Single-cell dynamics of Her6::Venus observed in progenitors at different stages in development. Related to Fig 2.

A, B Representative examples of cells classified as oscillatory (A) and non-oscillatory (B) showing corresponding single-cell time series of Her6::Venus (panel 1), mKeima-H2B (panel 2), Her6::Venus/H2B (panel 3) and detrended Her6::Venus/H2B (panel 4); statistics denote log-likelihood ratio (LLR) (A, B—panel 4) and period per cell (A—panel 4). Data were collected from 3 embryos with 1 embryo per condition corresponding to the developmental stages 28, 30 and 34 hpf (indicated in panel 1).

 © 2020 The Authors

 © 2020 The Authors

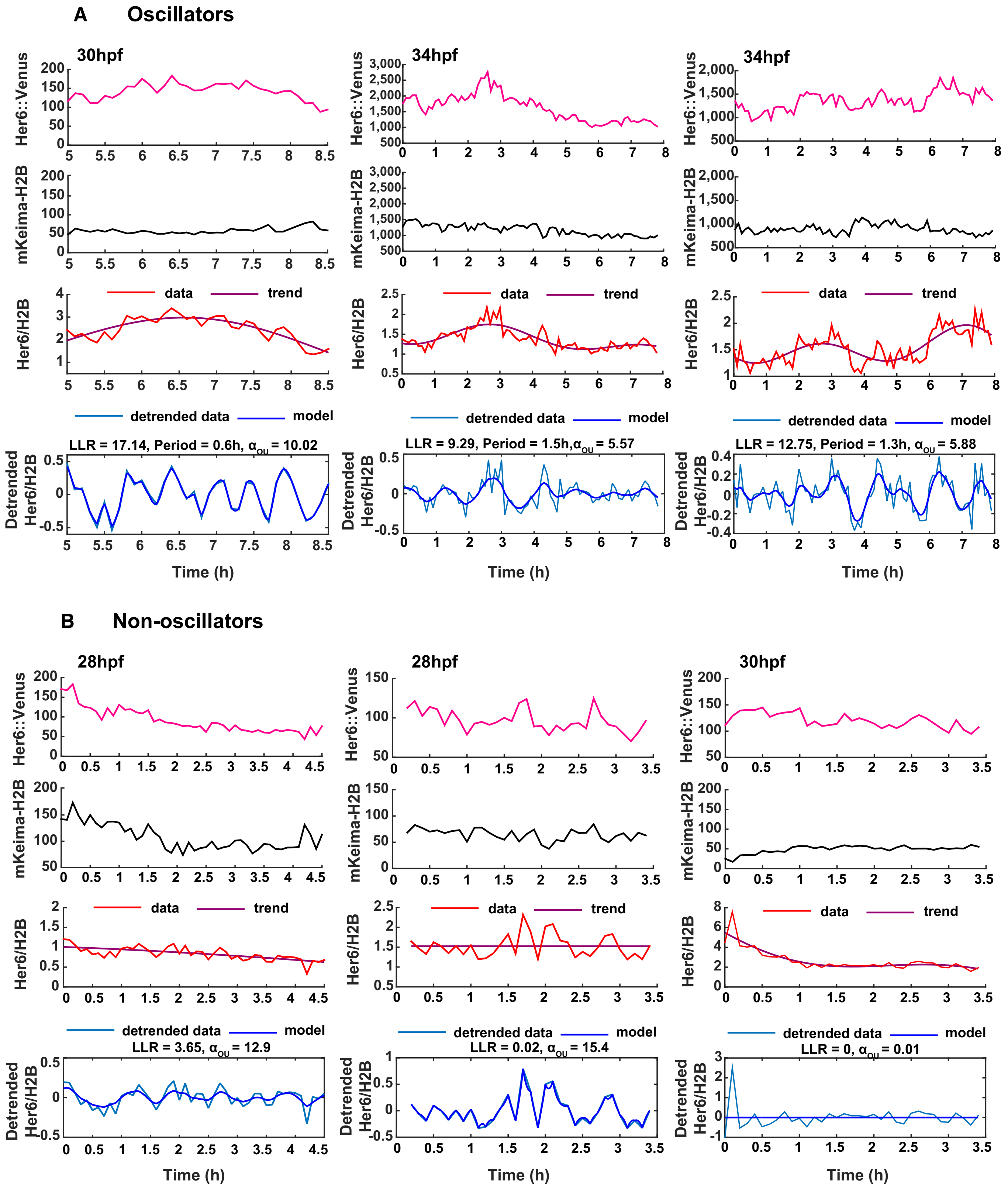
A Oscillators
30hpf
34hpf
34hpf
Her6::Venus
mKeima-H2B
Her6/H2B
data
trend
detrended data
model
LLR = 17.14, Period = 0.6h, α_OU = 10.02
LLR = 9.29, Period = 1.5h,α_OU = 5.57
LLR = 12.75, Period = 1.3h, α_OU = 5.88
Detrended Her6/H2B
Time (h)
B Non-oscillators
28hpf
28hpf
30hpf
LLR = 3.65, α_OU = 12.9
LLR = 0.02, α_OU = 15.4
LLR = 0, α_OU = 0.01

Figure EV1.

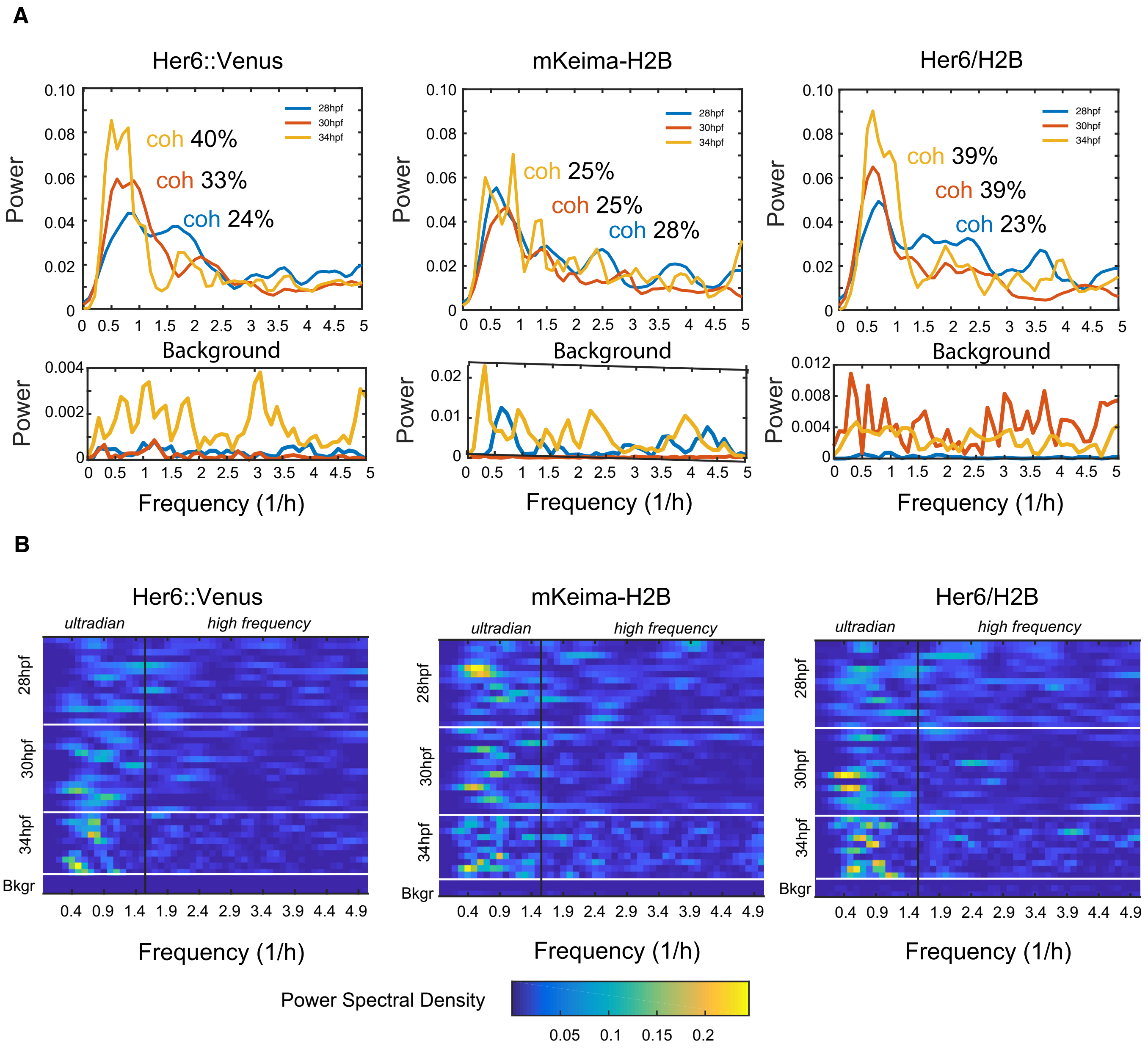

Figure EV2. Power analysis of Her6::Venus in progenitors at different stages in development. Related to Fig 2.

A (A—top panels) Power spectrum reconstructions showing population averages of single cell spectra computed from the detrended Her6::Venus, mKeima-H2B nuclear marker and Her6::Venus/H2B timeseries and comparative values for coherence observed at population level at 28, 30 and 34 hpf developmental stages; (A—bottom panels) aggregate power spectra for technical white noise (labelled as Background) collected from areas of the tissue that do not express the fluorophore.

B Heatmap representation of single cell power spectral density (PSD) observed in detrended Her6::Venus, mKeima-H2B nuclear marker and Her6::Venus/H2B timeseries at different stages with example Background (Bkgr) traces indicative of technical white noise; data correspond to aggregate measures shown in (A); frequency ranges corresponding to ultradian periodicity and high frequency are delineated. Data consist of 28 hpf (14 cells, 1 embryo), 30 hpf (14 cells, 1 embryo) and 34 hpf (10 cells, 1 embryo) collected from three independent experiments.

© 2020 The Authors

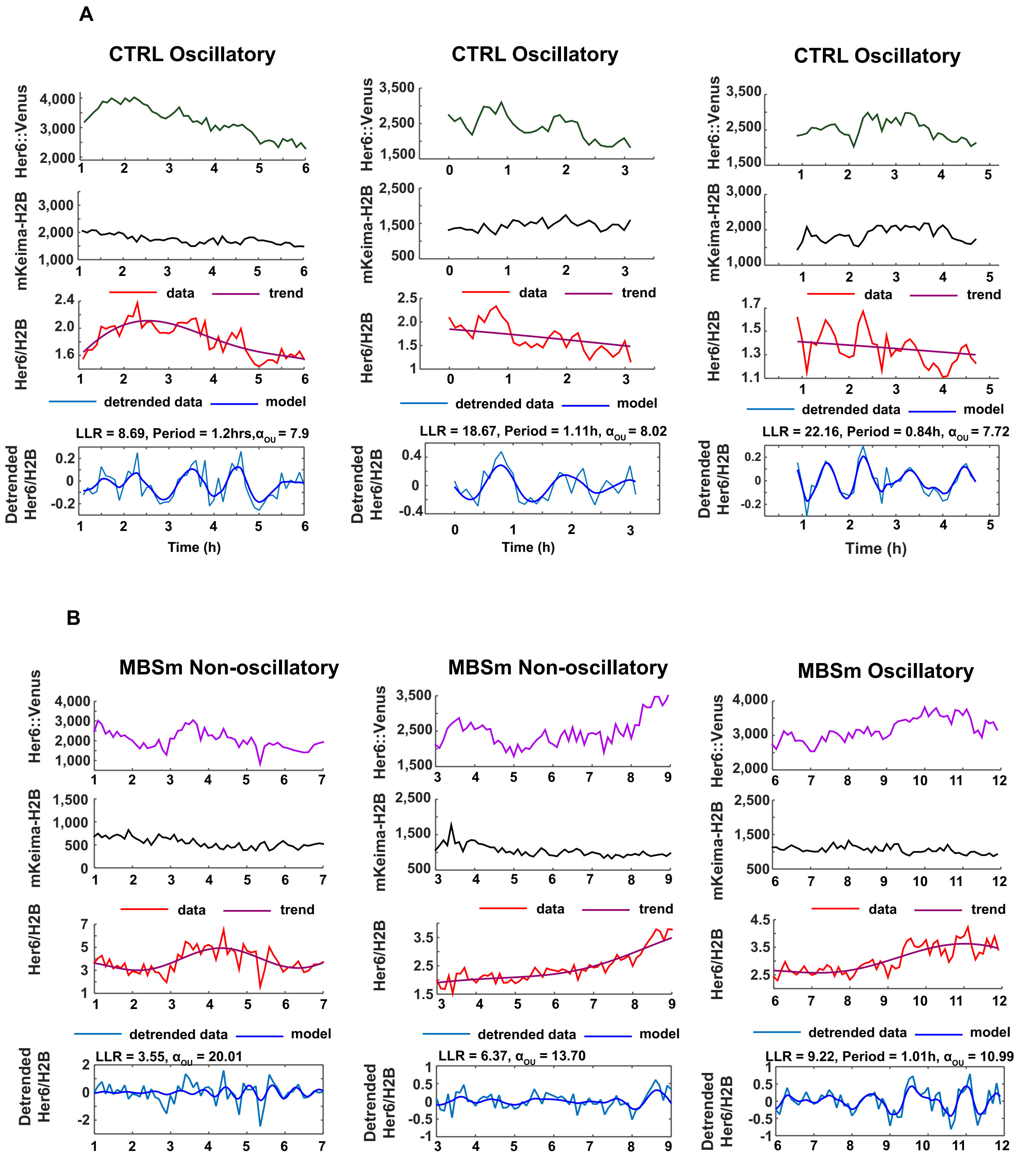

Figure EV3. Single cell dynamics of Her6::Venus observed in progenitors in the presence and absence of miR-9 regulation. Related to Fig 4.

A, B Representative examples of time series collected from control embryos (A—CTRL) and embryos containing a mutation of the miR-9 binding site (B—MBSm). Panels include corresponding single cell time series of Her6::Venus (panel 1), mKeima-H2B (panel 2), Her6::Venus/H2B (panel 3) and detrended Her6::Venus/H2B (panel 4); statistics in panel 4 indicate log-likelihood ratio (LLR) and period for cells classified as oscillatory and LLR values for non-oscillatory cells, as well as aperiodic lengthscale (α_{OU}) values. Data show examples collected from 4 different embryos with 2 embryos per condition corresponding to developmental stage 34 hpf.

© 2020 The Authors

Figure EV4. Frequency analysis of Her6::Venus observed in progenitors in the presence and absence of miR-9 regulation. Related to Fig 4.

A, B Representative example of power spectrum reconstruction showing corresponding data from Her6::Venus/H2B and mKeima-H2B detrended time series collected from one control (CTRL) embryo and one miR-9 binding site mutated (MBSm) embryo imaged simultaneously starting from 34 hpf; data represent aggregate power average of CTRL (15 cells, 1 embryo) and MBSm (14 cells, 1 embryo); power spectra from technical white noise labelled as Background show average of 2–3 timeseries collected in the absence of the fluorophore.

C Comparative analysis of coherence values observed in CTRL and MBSm embryos using power spectrum analysis that includes examples in (A,B); dots indicate coherence per embryo calculated from 4 embryos per condition consisting of CTRL (15 cells, 5 cells, 13 cells, 7 cells; 4 embryos) and MBSm (14 cells, 5 + 12 cells, 4 + 8 cells, 13 cells; 6 embryos); bars indicate mean and SD; unpaired *t*-test with two-tailed significance for $^{*}P < 0.05$, $^{**}P < 0.01$.

D, E Heatmap representation of single cell power spectral density (PSD) observed in Her6::Venus/H2B and mKeima-H2B timeseries corresponding to aggregate measures included in (A and B); PSD of technical white noise labelled as Bkgr showing one timeseries per condition; vertical lines delineate ultradian periodicity from high frequency.

F Left: Quantification of the contribution of high-frequency noise to single cell PSD shown in (D, Her6::Venus/H2B); bars represent median and interquartile range of 14–15 cells per embryo per condition; statistical test represents Mann–Whitney with two-tailed significance $^{**}P < 0.01$. Right: Quantification of high-frequency contribution with dots indicating median from 4 paired CTRL versus MBSm experiments with 1 embryo per condition (sample sizes stated in C); statistical tests indicate paired *t*-test with two-tailed significance $^{**}P < 0.01$.

G Pooled analysis of high-frequency contribution quantified from Her6::Venus/H2B and H2B in CTRL (50 cells, 4 embryos) and MBSm (56 cells, 6 embryos) also including data shown in (F); bars indicate median with interquartile range; statistical tests indicate Kruskal–Wallis with Dunn's multiple comparison test with significance $^{*}P < 0.05$, $^{**}P < 0.01$, $^{***}P < 0.001$.

© 2020 The Authors

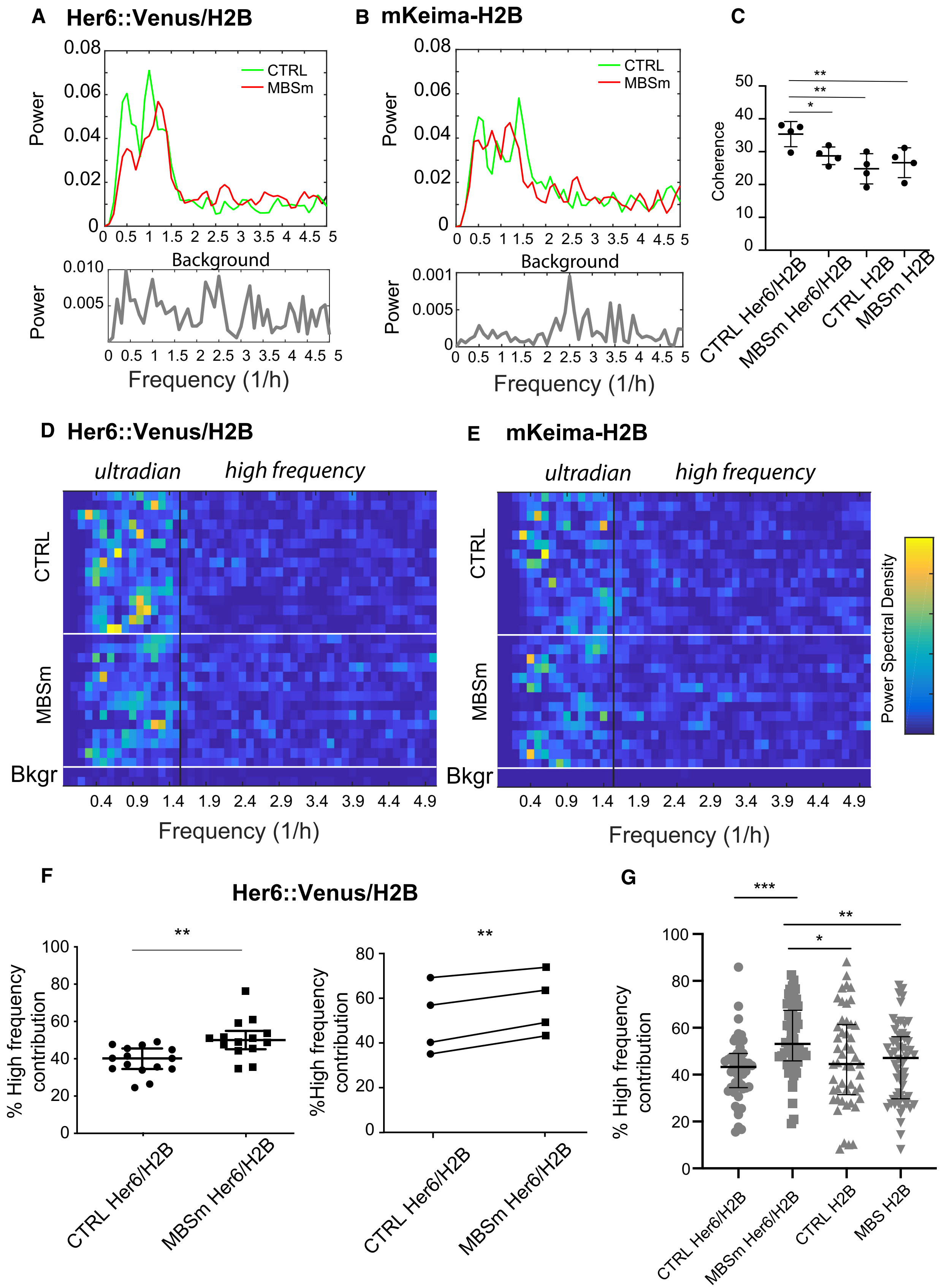
A Her6::Venus/H2B
Power
CTRL
MBSm
Background
Frequency (1/h)
B mKeima-H2B
Power
CTRL
MBSm
Background
Frequency (1/h)
C
Coherence
CTRL Her6/H2B
MBSm Her6/H2B
CTRL H2B
MBSm H2B
D Her6::Venus/H2B
ultradian
high frequency
CTRL
MBSm
Bkgr
Frequency (1/h)
E mKeima-H2B
ultradian
high frequency
CTRL
MBSm
Bkgr
Frequency (1/h)
Power Spectral Density
F
Her6::Venus/H2B
% High frequency contribution
CTRL Her6/H2B
MBSm Her6/H2B
%High frequency contribution
CTRL Her6/H2B
MBSm Her6/H2B
G
% High frequency contribution
CTRL Her6/H2B
MBSm Her6/H2B
CTRL H2B
MBS H2B

Figure EV4.
