# Supplementary material for: Dynamic properties of noise and Her6 levels are optimized by miR‐9, allowing the decoding of the Her6 oscillator
Source: EMBO J. 2020 May 12;39(12):e103558. doi: 10.15252/embj.2019103558 (PMC7298297; doi:10.15252/embj.2019103558)
Supplement: Supplementary file 6 — Source Data for Appendix [file EMBJ-39-e103558-s006.zip › embj2019103558-sup-0005-SDataFigS2.pdf]

Date

Notebook no.

Continued from page

Title

One to One meeting 9.5.16

black arrows indicate lanes used for the Figur S2c,

CRISPR18 indicates Knock-in 1 and CRISPR22 indicates Knock-in 2
+ indicates positive knock-in embryo by Cromogenic WMISH
- indicates negative knock-in embryo by Cromogenic WMISH

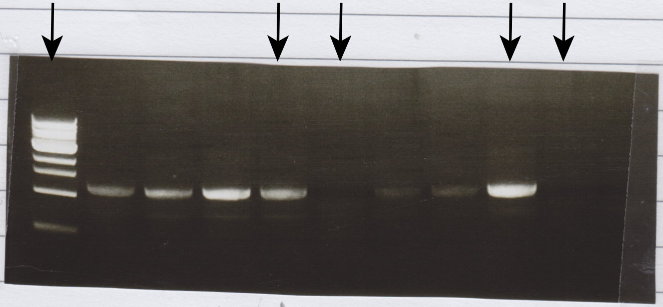

\+ + + + − + + + − ← 11 CRISPR

cromogenic CRISPR 18 — cromogenic CRISPR22.

primers 800/215

Tm 54

15

→ 1200bp

200
